# Draft Genome Sequence of Uncultivated Desulfosporosinus sp. Strain Tol-M, Obtained by Stable Isotope Probing Using [^13^C_6_]Toluene

**DOI:** 10.1128/genomeA.01422-14

**Published:** 2015-01-15

**Authors:** Nidal Abu Laban, BoonFei Tan, Anh Dao, Julia Foght

**Affiliations:** Department of Biological Sciences, University of Alberta, Edmonton, Alberta, Canada

## Abstract

A draft Desulfosporosinus genome was assembled from the metagenome of a methanogenic [^13^C_6_]toluene-degrading community. The Desulfosporosinus sp. strain Tol-M genome is distinguished from that of previously published Desulfosporosinus strain by containing *bss*, *bbs*, and *bam* genes encoding enzymes for anaerobic biodegradation of monoaromatic hydrocarbons and lacking *dsrAB* genes for dissimilatory sulfate reduction.

## GENOME ANNOUNCEMENT

Monoaromatic hydrocarbons including benzene, toluene, ethylbenzene, and xylenes (BTEX) are major environmental pollutants that are difficult to remove from contaminated sites due to their toxicity and relative recalcitrance under anaerobic conditions. The spore-forming bacterial genus Desulfosporosinus is frequently detected in anaerobic hydrocarbon-impacted environments and is thought to play a role in the anaerobic biodegradation of BTEX compounds under sulfate-reducing conditions (1–3). Additionally, recent DNA–stable isotope probing (DNA-SIP) studies revealed the importance of Desulfosporosinus members in methanogenic toluene degradation (4, 5). Of the eight described Desulfosporosinus species, one of which degrades toluene (D. youngiae DSM 17734^T^ CM001441) (6), four complete genome sequences have been published (7), yet genes known to be involved in hydrocarbon activation have not been annotated in those species. Here, we provide a draft genome of an uncultivated Desulfosporosinus that differs from published genomes by containing genes for anaerobic toluene activation via benzylsuccinate synthase (*bssABC*) but lacking genes for sulfate reduction (*dsrAB*).

The draft genome (herein named Desulfosporosinus sp. Tol-M) was obtained by [^13^C_6_]toluene DNA-SIP of a methanogenic enrichment culture derived from oil sands tailings (8). Total DNA was isolated, and fractionated, and the ^13^C “heavy DNA” fraction was sequenced using Illumina MiSeq (http://tagc.med.ualberta.ca). Metagenomic reads were subjected to quality control and *de novo* assembly using CLC Genomics Workbench (CLC-Bio, USA). Scaffolds associated with the family Peptococcaceae were binned from the metagenome using sequence composition- and homology-based methods and subjected to decontamination as in (9). The genomic bin was annotated using RAST (http://rast.nmpdr.org).

The draft genome 16S rRNA gene had 96% identity to Desulfosporosinus meridiei DSM 13257 (AF076527), placing the 16S rRNA gene within the Desulfosporosinus clade. However, the average nucleotide identity to other Desulfosporosinus genomes (7) was only 78 to 80% (comparing 355 to 574 fragments with a 1-kb cutoff). The genome size is ~3.0 Mb contained in 206 scaffolds (of 1,000 to 131,760 bp) with 44% GC content. The genome contained 107 of 110 single copy genes (10), with best BLASTP hits to Desulfosporosinus sequences. Annotation in RAST revealed 53 RNA genes and 3,001 protein-coding sequences assigned to 335 SEED subsystem categories. Genes for anaerobic hydrocarbon activation were located in clusters, including *bssABC* for activation of toluene by fumarate addition, *bbsBDEF* for beta-oxidation of benzylsuccinate, and *bamA-I* genes encoding components postulated to be involved in benzoyl-CoA dearomatization in anaerobes (11), confirming its genetic potential for BTEX degradation. However, unlike other reported Desulfosporosinus species, the draft genome lacks the canonical genes specific to dissimilatory sulfate reduction, such as *dsrAB*. This absence may explain its presence in a methanogenic community as a syntrophic toluene-degrader. Our repeated attempts to cultivate this organism were unsuccessful because it did not grow with sulfate as electron acceptor and required syntrophic partner(s) under methanogenic conditions. Therefore, this draft DNA-SIP genome provides insight into the importance and function of Desulfosporosinus sp. Tol-M in toluene degradation in the laboratory and in methanogenic environments.

### Nucleotide accession numbers.

The sequences from the whole-genome shotgun project investigating Desulfosporosinus sp. Tol-M have been deposited at DDBJ/EMBL/GenBank under accession number JQID00000000. The version described in this paper is the first version JQID01000000.
